# Design and implementation of GRIP: a computerized glucose control system at a surgical intensive care unit

**DOI:** 10.1186/1472-6947-5-38

**Published:** 2005-12-19

**Authors:** Mathijs Vogelzang, Felix Zijlstra, Maarten WN Nijsten

**Affiliations:** 1 Surgical Intensive Care Unit, Department of Surgery, University Medical Center Groningen, University of Groningen, Groningen, The Netherlands; 2 Department of Cardiology, Thoraxcenter, University Medical Center Groningen, University of Groningen, Groningen, The Netherlands

## Abstract

**Background:**

Tight glucose control by intensive insulin therapy has become a key part of critical care and is an important field of study in acute coronary care. A balance has to be found between frequency of measurements and the risk of hypoglycemia. Current nurse-driven protocols are paper-based and, therefore, rely on simple rules. For safety and efficiency a computer decision support system that employs complex logic may be superior to paper protocols.

**Methods:**

We designed and implemented GRIP, a stand-alone Java computer program. Our implementation of GRIP will be released as free software. Blood glucose values measured by a point-of-care analyzer were automatically retrieved from the central laboratory database. Additional clinical information was asked from the nurse and the program subsequently advised a new insulin pump rate and glucose sampling interval.

**Results:**

Implementation of the computer program was uneventful and successful. GRIP treated 179 patients for a total of 957 patient-days. Severe hypoglycemia (< 2.2 mmol/L) only occurred once due to human error. With a median (IQR) of 4.9 (4.2 – 6.2) glucose measurements per day the median percentage of time in which glucose fell in the target range was 78%. Nurses rated the program as easy to work with and as an improvement over the preceding paper protocol. They reported no increase in time spent on glucose control.

**Conclusion:**

A computer driven protocol is a safe and effective means of glucose control at a surgical ICU. Future improvements in the recommendation algorithm may further improve safety and efficiency.

## Background

Critically ill patients often suffer from 'stress hyperglycemia', a condition in which insulin resistance due to increased catecholamine levels causes high blood glucose values [1]. The association between stress hyperglycemia and adverse outcome has been observed in numerous patient categories, ranging from patients admitted to the general ward [2] to myocardial infarction [3] and stroke patients [4]. For decades, stress hyperglycemia was thought to be merely a marker of disease, and was tolerated as long as glucose levels were not excessively high (e.g., over 11.0 mmol/L). The publication of the Leuven intensive insulin therapy study in September 2001 caused a paradigm shift in critical care medicine [5]. This study showed that rapid lowering of blood glucose levels below 6.1 mmol/L and subsequent maintaining of normoglycemia reduce mortality and morbidity markedly. These results were confirmed by a before-after study performed by Krinsley, in which also a decrease in mortality was achieved with tight glucose control [6]. Especially for septic patients, guidelines now recommend using insulin to reduce high glucose levels [7]. However, infusing insulin in order to lower glucose levels bears the risk of inducing life-threatening hypoglycemia, especially in sedated patients admitted to an intensive care unit (ICU). In order to cut back this risk, glucose levels must be frequently measured. Each measurement calls for a decision on what action to take to keep glucose levels in the normal range. With recommended sampling frequencies ranging from every 1 – 2 hours to every 6 hours, implementation of tighter glucose control poses an important logistic challenge. Many investigators have proposed nurse-driven protocols for glucose control. After each glucose measurement, simple if-then rules or lookup tables yield an advice on how much insulin needs to be administered [8-10]. Even though glucose sampling frequency is high, reduction of hyperglycemia is often not satisfactory, and, more important, hypoglycemia is relatively common [9]. Glucose metabolism is also an important topic in acute coronary care. Several studies have evaluated glucose-related therapies as strategies to improve outcome in acute coronary syndromes, such as high dose glucose-insulin-potassium (GIK) infusion [11,12], or combined glucose-insulin infusion to reduce glucose levels [13,14]. Although clinical results have been mixed, with most recent results being negative, the efficacy of glucose-lowering interventions in acute coronary care is still unknown since none of the published trials achieved tight glycemic control [11,14]. Because coronary care units (CCU) are a less controlled environment with a lower personnel-to-patient ratio than ICUs, intensive insulin therapy is hard to achieve with paper protocols [14]. An optimized decision making algorithm might be able to make tighter control possible.

We hypothesized that a computer program can employ the necessary complex logic to achieve the desired level of both safety and efficiency of glucose control without excessive glucose sampling frequencies. In the beginning of 2003 we initiated development of a computer controlled decision support system.

## Methods

### Design rationale

**Figure 1:**
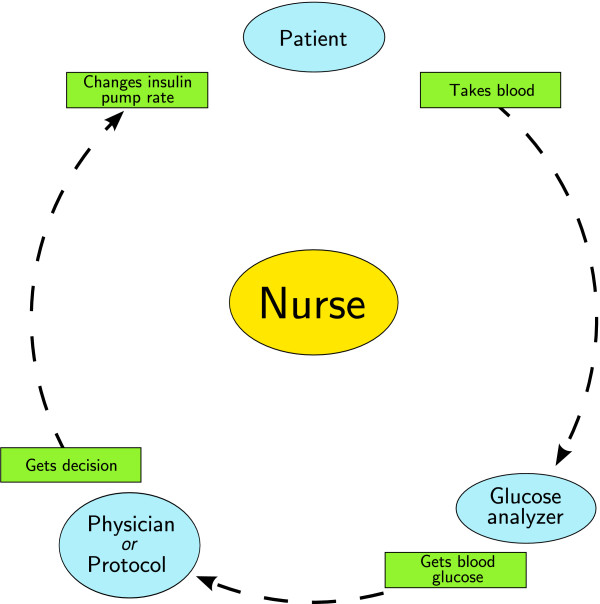
**Glucose control cycle**. Nurse-driven glucose control consists of the repetitive execution of the cycle depicted in this figure. First, the nurse acquires blood from the patients and gets the glucose level. A protocol or doctor then decides what action should be taken (in most cases a change of the rate of the insulin pump), and subsequently this action is performed at the bedside by the nurse.

We conceived the computer decision support system (CDSS) to be used primarily by nurses. Our first implementation site was our 12-bed surgical ICU at a tertiary teaching hospital, and we planned for future implementation at other ICUs and at the CCU. A thorough analysis of factors which lead to successful CDSS deployment and usage has previously shown integration of the system into routine clinical workflow to be of paramount importance [15]. Automatic provision of a concrete recommendation instead of merely an assessment of the situation, and provision of decision support at the time and place of decision making were other elements that were highly predictive of successful implementation. Figure 1 shows the different steps a nurse takes when performing glucose control. Repetitive glucose measurements are followed by appropriate action to maintain normoglycemia. At our surgical ICU, as well as at the CCU, blood glucose values were determined by a point-of-care blood gas analyzer, giving a very short delay between blood sampling and availability of the glucose value (~2 minutes). As most nurses wait at the point-of-care analyzer until the result of the analysis is known, a computer situated next to the analyzer was the ideal spot for the system to interact with the nurses and to give its recommendations. This ensured an easy integration in standard clinical workflow, and made sure that the recommendations were provided to the responsible nurse at the place and time when needed.

The project was named GRIP (Glucose Regulation for Intensive care Patients). One of the key design principles was that the output should be regarded as recommendations, not as orders. The recommendations that GRIP gives could be overruled at any time. If overruled, GRIP continues to give meaningful advice the next time (as long as it knows that its recommendation was not followed, and what action was taken instead). In this way, GRIP remains usable in various unforeseen situations.

### Technical design

**Figure 2:**
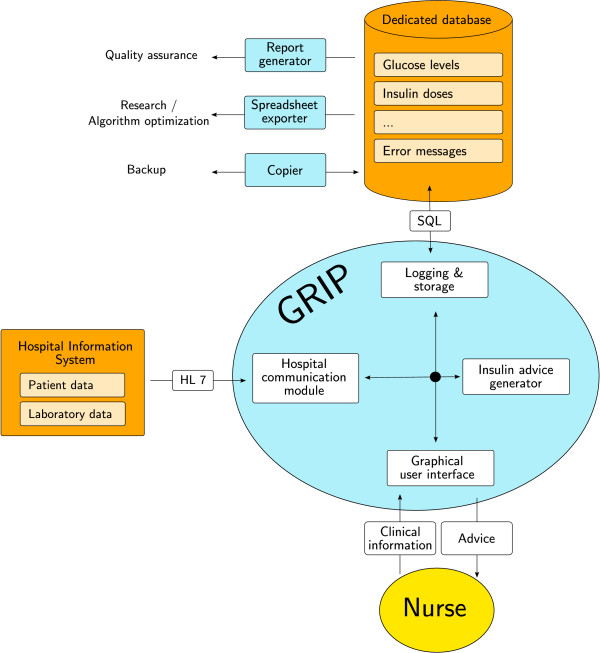
**High-level overview of GRIP**. Grip contains four major components: a component to interface with the hospital information system, a component to interface with the nurse, a component that calculates the advice that GRIP gives, and a component that logs errors and stores all data GRIP generates. HL7 : Health Level 7. SQL : Standard Query Language.

A high-level overview of GRIP's design is shown in figure 2. To enhance safety and facilitate user acceptance, all information that was available in the central hospital database was queried from there and not from the nurse. To ensure correct glucose values, each glucose value that was retrieved from the laboratory system required a validation from the responsible nurse. In this way, measurement errors, patient swaps, and other mistakes were prevented from disturbing the decision making of GRIP. After each glucose measurement, a number of clinical variables were queried from the nurse (table 1). The communication with the hospital database was designed using standard communication protocols such as Health Level 7 (HL7). Data collected and computed by GRIP, as well as error messages, were stored in a relational database using standard query language (SQL) queries. Being in a database system, the data was easily accessible for special-purpose applications, such as a report generator for periodic auditing of quality of glucose control or other aspects of GRIP, or for a data extraction program for purposes of research. Most data in the database was stored as pieces of eXtensible Markup Language (XML), which facilitates extension of data storage in the future.

**Table 1:** Data input into GRIP

	From the hospital database	From the nurse
Patient characteristics (once at admission)	Name Birth-date Admitting department Hospital unique patient ID	Reason of admission History of diabetes Length Weight
For every glucose measurement	Glucose value	Nurse identification Enteral glucose dose Stomach retention Intravenous glucose dose Current insulin pump rate Mean arterial pressure > 70 (yes/no) Noradrenalin dose Dopamin dose Steroid administration (yes/no)

Variables that GRIP currently queries from the central hospital database or from the nurses.

### Recommendation generation algorithm

Recommendations generated by GRIP mainly consist of two parameters: the recommended insulin pump rate and the time at which the next blood sample should be taken. Also, in special cases (currently on occurrence of glucose values > 15 or < 4 mmol/L) the recommendation is accompanied by a request to consult the attending physician. On low glucose levels (< 3 mmol/L) the 'hypoglycemia advice' is given, which consists of an intravenous glucose dose, an insulin pump rate of zero, and prompt notification of the attending physician. Our current implementation of GRIP is based on the use of continuous insulin infusion and does not recommend boluses of insulin. It has been observed that due to a saturation effect, continuously infused insulin is more effective than boluses of insulin [16].

We will briefly describe the algorithm for pump rate. We hypothesized that by using solely the most recent glucose value, efficient glucose control is hard, if not impossible, to achieve, and chose to include rate of change of glucose as secondary parameter. The basis for our pump rate algorithm is the following formula:

Δ*I *= (1 + 0.25·I−4h¯MathType@MTEF@5@5@+=feaafiart1ev1aaatCvAUfKttLearuWrP9MDH5MBPbIqV92AaeXatLxBI9gBaebbnrfifHhDYfgasaacH8akY=wiFfYdH8Gipec8Eeeu0xXdbba9frFj0=OqFfea0dXdd9vqai=hGuQ8kuc9pgc9s8qqaq=dirpe0xb9q8qiLsFr0=vr0=vr0dc8meaabaqaciGacaGaaeqabaqabeGadaaakeaadaqdaaqaaiabdMeajnaaBaaaleaacqGHsislcqaI0aancqWGObaAaeqaaaaaaaa@3142@)·(0.2·(*G*_0 _- *G*_*target*_) + 0.3·Δ_-4*h*_*G*)

In this formula, Δ*I *is the proposed change in pump rate, I−4h¯
 MathType@MTEF@5@5@+=feaafiart1ev1aaatCvAUfKttLearuWrP9MDH5MBPbIqV92AaeXatLxBI9gBaebbnrfifHhDYfgasaacH8akY=wiFfYdH8Gipec8Eeeu0xXdbba9frFj0=OqFfea0dXdd9vqai=hGuQ8kuc9pgc9s8qqaq=dirpe0xb9q8qiLsFr0=vr0=vr0dc8meaabaqaciGacaGaaeqabaqabeGadaaakeaadaqdaaqaaiabdMeajnaaBaaaleaacqGHsislcqaI0aancqWGObaAaeqaaaaaaaa@3142@ is the mean pump rate over the 4 hours preceding the last glucose measurement, *G*_0 _is the most recent glucose value, *G*_*target *_is the target glucose value, and Δ_-4*h*_*G *is the change in glucose level between the last glucose value and the value 4 hours earlier (this value is linearly interpolated). The first term of the formula ensures that when a patient receives a high insulin dose, the change the algorithm suggests will be larger than when the current dose is low. The formula exhibits two interesting properties. First, the algorithm needs an explicit target blood glucose level to aim for. As the optimal blood glucose target is still subject to discussion, we have set this value to 6.5 mmol/L [17,18]. Changing the target to a different value would be easy to accomplish. Second, the algorithm only uses data from the most recent four hours. As a consequence, potentially valuable preceding data are ignored and oscillation of glucose levels may be induced by the short time interval. However, we decided in favor of this short interval because of its robustness to sudden changes. In case of a sudden increase or decrease in insulin sensitivity due to some clinical event unknown to GRIP, the preceding glucose and insulin values become less relevant, or in the worst case even misleading to the recommendation algorithm. Therefore, provided glucose is sampled sufficiently often, the short lookback time makes quick adaptation to new situations possible. In our implementation, the result of the aforementioned formula is only an intermediate result. Decreases in enteral or intravenous glucose administration as filled out by the user automatically lead to a proportional reduction of the dose recommendation, e.g., if the patient received 64 ml/hour of enteral feeding and this is lowered to 16 ml/hour, the advised pump rate will, depending on other factors, be decreased by approximately three quarters. To improve user acceptance, recommendations lower than 0.3 units/hour are converted to 'no pump'. The final recommendation is generated after applying a number of safety measures, the most important being the limitation that GRIP never recommends a pump increase of more than 1.5 units/hour and never recommends pump rates over 10 units/hour. Saturation of the effect of insulin provides another argument for limiting the pump rate [16,19].

The algorithm that calculates the desired time of the next glucose measurement is considerably more complex. Its main feature is a calculated measure conceived to quantify the risk of hypoglycemia. The following measures lead to a higher predicted risk of hypoglycemia, and consequently to a shorter interval to the next advised glucose sample: high insulin pump rate, high recommended pump increase, fast glucose decrease (as measured by the extrapolated glucose in 4 hours, derived from the current glucose value and the current ΔglucoseΔt
 MathType@MTEF@5@5@+=feaafiart1ev1aaatCvAUfKttLearuWrP9MDH5MBPbIqV92AaeXatLxBI9gBaebbnrfifHhDYfgasaacH8akY=wiFfYdH8Gipec8Eeeu0xXdbba9frFj0=OqFfea0dXdd9vqai=hGuQ8kuc9pgc9s8qqaq=dirpe0xb9q8qiLsFr0=vr0=vr0dc8meaabaqaciGacaGaaeqabaqabeGadaaakeaadaWcaaqaaiabfs5aejabbEgaNjabbYgaSjabbwha1jabbogaJjabb+gaVjabbohaZjabbwgaLbqaaiabfs5aejabbsha0baaaaa@3A8E@), low or decreasing glucose intake, and occurrence of recent low glucose values. The maximum advised glucose sampling interval is 12 hours, and the minimum interval is 30 minutes.

### User interface

**Figure 3:**
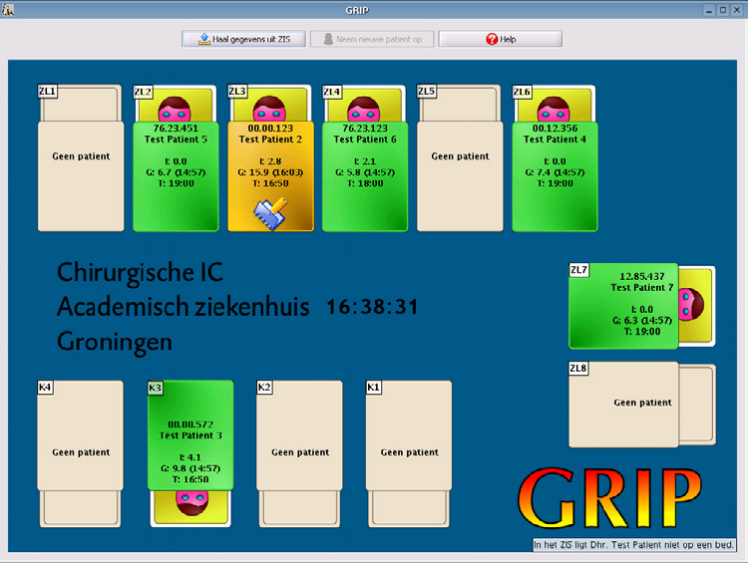
**Main screen of GRIP**. The main screen of GRIP. An overview of the ICU is shown replicating the arrangements of beds on the floor. Beds have colors according to pending action: green – no action has to be taken, orange – action has to be taken and a small icon indicates what action, in this case a new glucose value was detected in the hospital data system, which needs a validation from the nurse, and red – urgent action required, for example occurrence of hypoglycemia, or an advised measurement that is more than 30 minutes late. Each bed shows the current insulin pump rate, the most recent glucose value and the time it was taken, and the time the next glucose value needs to be taken. Empty beds are shown in grey. Each bed is clickable to yield a more detailed information panel shown in figure 4.

**Figure 4:**
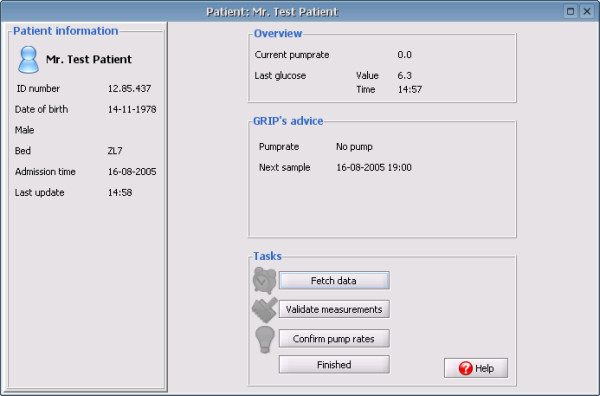
**Patient overview in GRIP**. The patient overview panel shows more detailed information for a single patient. On the left general characteristics such as patient ID number, birth-date and sex are shown. The middle three panels show the current status, the recommendation of GRIP, and the tasks GRIP thinks the user should perform.

A graphical user interface was designed to both acquire clinical information from the nurse and communicate advice to the nurse. To make user input as simple as possible, all filled out information is automatically filled in with the previous values so that only changes require user action. Figure 3 shows the main screen of GRIP. This screen provides a quick overview of all patients. Figure 4 shows the screen providing details for one patient. The required action which a nurse should take was divided in three steps:

1. Take a blood sample and analyze it. Clicking the button in GRIP makes GRIP query the hospital information system to see if it can retrieve the new value.

2. Review the current information of a patient and update this information if changes have occurred. This step also includes validating each newly acquired glucose measurement.

3. Review the insulin pump advice given by GRIP and tell GRIP whether it was accepted and executed or, if not, what the new insulin pump rate is.

These three steps normally follow each other in order, but each step is also immediately accessible without following the previous steps (e.g., a physician who changes an insulin pump could immediately tell this to GRIP by taking step 3, without the need to first take blood, etc.)

### Hardware and availability

The program was designed to run on a standard commodity personal computer, situated directly next to the point-of-care glucose analyzer for optimal integration in the clinical workflow. The program was developed in Java, a freely available multi-platform language. Because MySQL^® ^(a freely available open-source database server) was chosen as the database system, the entire system can be run using only free software. Upon completion of a new feature-complete version scheduled for the beginning of 2006, GRIP will be released under the open source Gnu Public License [20], and will be downloadable from . This means GRIP can be used by anyone free of charge.

### Evaluation of the system

Glucose control before implementation of GRIP was performed by a sliding-scale paper protocol. This protocol advised an insulin pump rate which was based on the last measured glucose level. Measurements were taken 4 times a day at a fixed schedule. When needed, the scheme was adjusted for individual patients on a daily basis. The medical staff of the ICU was informed and unanimously agreed to gradually implement GRIP. Since the move from conventional practice to GRIP constituted an organizational and logistic change that aimed to improve existing and accepted medical practice, approval by the medical ethical committee or patient consent was deemed unnecessary. To get a basic idea of control achieved by the system, we recorded all glucose measurements of all patients during the first 4 month period in which GRIP was in effect. We also retrieved age, reason of admission, history of diabetes, APACHE II score, and both ICU and hospital mortality for these patients. Safety of control was assessed by checking all patients for occurrences of severe hypoglycemia (< 2.2 mmol/L) or mild hypoglycemia (< 3.5 mmol/L). To assess the ability to reduce hyperglycemia, all patients staying longer than 24 hours were analyzed. As the target level was 6.5 mmol/L, we defined a target glucose range of 4–7.5 mmol/L. For each patient, we determined how much time the glucose levels were within this target range and we represented this value as the fraction of the entire length of stay. We determined the time from admission to the first glucose under 7.5 mmol/L, and calculated median and interquartile range for glucose levels at 6-hour intervals for the first two days. Finally the hyperglycemic index, a measure indicative of overall hyperglycemia that is not lowered by hypoglycemic episodes, was calculated for each patient [21].

Patients at the ICU were enterally fed as soon as possible. When enteral feeding was not possible for prolonged periods, total parenteral nutrition or concentrated glucose infusions were started at moderate doses (100–200 grams per day).

User acceptance was assessed by a questionnaire. Nurses were asked to fill out the questionnaire 1 month before and 6 months after implementation of GRIP. The questionnaire held 6 months after implementation of GRIP contained a number of questions asking for a direct comparison between working with GRIP and working with the paper protocol.

### Statistical analysis

For the nurse questionnaires, a 7 point scale of agreement was used. Differences before and after usage of GRIP were tested with the Mann-Whitney U test. All data are presented as median (interquartile range). Statistical analysis was performed using R version 2.1.0 [].

## Results

### Implementation details

Implementation of GRIP began in the beginning of 2003. In the fall of 2003 an initial version of GRIP was tested for three weeks at the surgical ICU without nurses or doctors following its recommendations. Nurse feedback indicated that this test version's user interface was insufficiently intuitive. In November 2004 the final version of GRIP, including the improved user interface as described in the methods, was deployed. First it was monitored for 6 weeks, while the regular paper protocol was still followed. In the next two weeks we started following GRIP's recommendations, gradually increasing its usage bed-by-bed. From January 2005 on, insulin therapy of patients on all beds was determined by GRIP. During the first weeks of deployment a number of small changes to solve trivial problems were needed. Thereafter, two major problems have occurred. First, the computer running GRIP experienced a sudden complete hard-disk crash in April 2005, losing all data. Using our automatic daily backup, the system was up and running again within 6 hours, with less than 24 hours data loss, which was easily reentered into GRIP from the charts. The second problem was the transition to daylight savings time. This was handled correctly by GRIP, but unfortunately the central hospital system only picked up the correct time later during the next day. Before the central system was adjusted to the proper time, all measurements appeared to GRIP one hour later than they actually were. Apart from inconvenience to the nurses, these problems caused no misleading recommendations or dangerous situations. Currently, the system has been running without interruption for over 3 months without any change, maintenance, crash or other problem. A number of other problems have occurred, all external to GRIP, which would have affected a paper protocol equally as much as it affected GRIP. These included measurements that failed to be included in the central hospital database and malfunction of the point-of-care analyzer. After these problems were resolved and GRIP started receiving correct data again, its recommendations could immediately be followed, because GRIP ignored the erroneous data.

### Training of nurses

Basic data entry in GRIP proved to be very straightforward. Only data entry of events that happened in the past was not completely obvious. During the first 2 month observation period, residents were asked to perform data entry and as no recommendations were used, they could experiment and save any questions until the lead programmer (MV) was present at the ICU. When real use of GRIP by nurses was initiated, almost all residents knew how to work with GRIP and could provide explanation to nurses. The staff intensivist (MN) involved in the design of GRIP also provided explanation when asked. No special meetings for training had to be scheduled.

### Safety and efficiency

**Figure 5:**
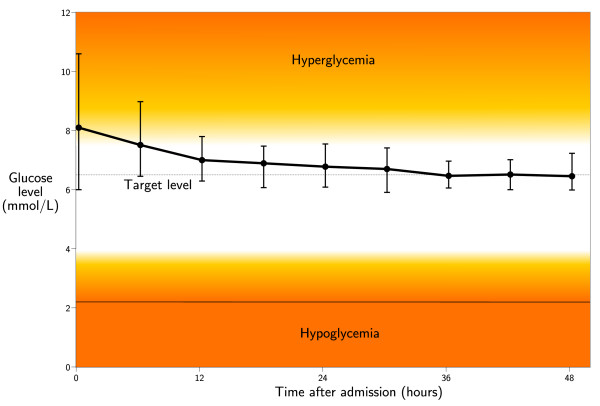
**Glucose control**. Median and interquartile range of glucose levels are shown for the 109 patients with a length of stay longer than 1 day. The dashed line equals the glucose target of GRIP.

During the 4-month period from January 1st to May 1st, 2005, 179 patients were treated at our ICU, for a total of 957 patient-days. Patient characteristics are shown in table 2. Severe hypoglycemia (< 2.2 mmol/L) occurred once in one patient (0.6 %). Analysis revealed that this was caused by a human error involving accidentally increasing the rate of the insulin pump. Mild hypoglycemia (< 3.5 mmol/L) occurred in 20 patients (11.2 %). 109 out of 179 patients stayed longer than one day at the ICU. Data regarding hyperglycemia in these patients is presented in table 3. Glucose levels met the target range for more than three-quarters of the time in 66 patients (61 %). The glucose sampling frequency was less than 6 times a day in 76 patients (70 %). The median hyperglycemic index indicates that the median patient had a time-weighted glucose level of 6.9 mmol/L. The maximum insulin dose of 10 units/hour was needed in 5 patients (5 %). At the time of maximum insulin infusion, the glucose values of these patients were 8.3, 8.8, 9.3, 14.9 and 17.6 mmol/L. Figure 5 shows median and interquartile range of glucose levels during the first 48 hours of ICU stay.

**Table 2:** Patient characteristics

N	179
Age	62 (51 to 72)
Male sex	109 (61 %)
Reason of admission	
Abdominal surgery	87 (49 %)
Vascular surgery	24 (13 %)
Trauma	17 (9.5 %)
Liver transplant	14 (7.8 %)
Miscellaneous	37 (21 %)
APACHE II	14 (11 to 19)
History of diabetes	26 (14.5 %)
Length of stay at the ICU (days)	1.6 (0.8 to 4.7)
Mortality at the ICU	19 (10.6 %)
Hospital mortality	26 (14.5 %)

Patient characteristics of all patients treated from January 1st to May 1st, 2005.

**Table 3:** Glycemic control

Time from admission to first glucose (hours)	0.7 (0.3 to 2.7)
Admission glucose (mmol/L)	8.1 (6.1 to 10.5)
Time to first glucose < 7.5 mmol/L (hours)	5.7 (1.2 to 11.4)
Glucose change in the first 24 hours (mmol/L)	-1.2 (-3.9 to +1.4)
Glucose level after 24 hours (mmol/L)	6.7 (6.0 to 7.5)
Fraction of time with glucose between 4 and 7.5 mmol/L	78 % (66 % to 88 %)
Hyperglycemic index (mmol/L)	0.96 (0.68 to 1.37)
Number of glucose samples per day	4.9 (4.2 to 6.2)

This table shows how hyperglycemia is treated in the 107 patients staying more than 1 day at the ICU. Data presented are median (IQR).

### User acceptance

**Figure 6:**
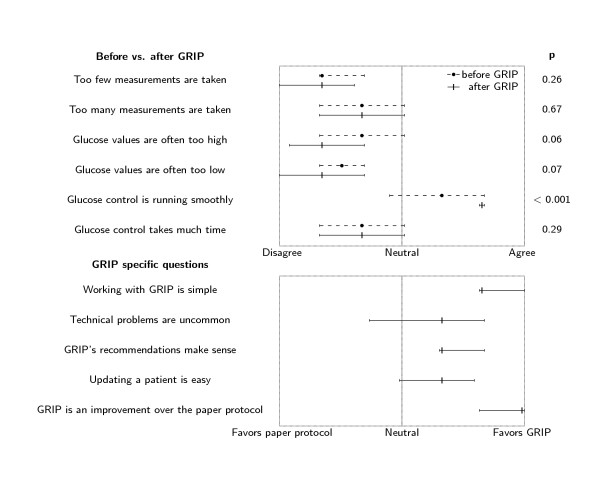
**Results of the nurse questionnaire**. Results of the nurse questionnaires 1 month before (N = 32) and 6 months after (N = 22) implementing GRIP. Questions pertaining to GRIP were only asked in the questionnaire held after 6 months. The median and interquartile range of responses are shown for each question asked.

Questionnaires were filled out by 32 nurses before implementation of GRIP and by 22 nurses after implementation. After deployment of GRIP, nurses judged glucose control to be running more smoothly than before deployment of GRIP (p < 0.001, figure 6). There was a trend towards nurses finding that glucose values are less often too high or too low. GRIP was judged as being simple to work with, and chosen almost unanimously as an improvement over the old paper protocol. Estimated time spent on glucose control per working shift was 10 (6 to 12) minutes before GRIP and 10 (10 to 15) minutes after GRIP (p = 0.13). Analysis of paired observations before and after implementation of GRIP revealed similar results. From the free text section of the questionnaire it was clear that the nurses found that GRIP was able to adequately manage glucose control in more patients than the paper protocol. Nurses therefore did not need to call the attending physician as often as before and thus were able to devote more time to other tasks.

## Discussion

In this report we present GRIP, a computer decision support system for glucose control by intensive insulin therapy. This system was successfully implemented at a surgical ICU, and was found to provide safe and efficient glycemic control. User acceptance, an important hurdle for successful implementation of a clinical decision support system, was excellent with minimal training. Nurses clearly rated GRIP as an improvement over the conventional sliding scale protocol. GRIP will be released under an open source license and will thus be free to use and improve by anyone in the future. We think especially GRIP's independence of a patient data management system (PDMS) is a strong point that makes it widely usable. At ICUs where a PDMS is already operational, GRIP might introduce double data entry. However, this can be easily resolved by either letting GRIP query the PDMS for information or by converting GRIP's advice module into a plug-in of the PDMS.

The efficiency and safety of control achieved by GRIP's current relatively simple algorithm was satisfactory. In the Leuven study, a mean glucose level of 5.7 mmol/L was achieved in a cohort of patients with a median APACHE II score of 9 [5]. Krinsley achieved a mean of 7.3 mmol/L in a sicker cohort of patients (median APACHE of 15) [6]. Our group had APACHE scores similar to Krinsley's, and a mean glucose level that was 0.4 mmol/L lower. With regard to hypoglycemia, in the Leuven study 39 out of 765 patients (5.1 %) had one or more values lower than 2.2 mmol/L, compared to 1 out of 179 (0.6 %) in our group. Krinsley describes the number of hypoglycemic episodes as the proportion of total number of glucose measurements, instead of as the proportion of patients at risk. In Krinsley's study, 0.34 % of measurements were lower than 2.2 mmol/L, compared to 0.02 % in our group.

During the design of the current recommendation algorithm a number of arbitrary decisions were taken. Based on experience, we chose to include glucose value and glucose difference as the two most important determinants of advised pump rate, and we chose to limit pump rate to 10 units per hour. Although evidence for the rationality of the latter decision exists [16,19], most decisions were taken purely based on prior experience in controlling glucose levels. Our results show that this algorithm gives satisfactory results, but we are aware that GRIP has the potential for substantial improvements in the future, as our choices are unlikely to be the most optimal. For example, the current algorithm is very cautious and only takes small steps when increasing the insulin pump rate, sacrificing rapid control in favor of safety. Analysis of the data that have been collected thus far may reveal situations in which the algorithm can safely take larger steps to achieve more timely glucose control. It will be interesting to analyze differences between groups of patients with respect to factors such as reason of admission, presence of diabetes, and concomitant drug use. This will allow glucose control to transition from the current general "one size fits all" approach, which uses the same advice for every patient, to a more tailor-made approach, which makes use of as much information as possible to make its advice fit an individual patient's needs. As the advice generation module can be changed without affecting the user interface, algorithm improvements can be implemented without additional training of the nurses.

Prior studies have evaluated different types of computer systems for glucose control in critically ill patients. A number of studies have evaluated feasibility of continuous glucose sensors, which in theory provide ultimate efficiency and safety of control. However, apart from the obvious extra costs, continuous sensors have been found to lack reliability, requiring frequent replacements of the sensor, and report inaccurate data in the hypoglycemic range [22,23]. We believe that for the foreseeable future, the vast majority of glucose control schemes will still employ sequential discontinuous measurements. Chase et al. have worked on developing models for accurate insulin dose prediction in critically ill patients, but with 2 measurements per hour, measurement frequency was much higher than GRIP's [19,24]. Rood and colleagues have previously investigated computerizing guidelines for glucose control and found that glucose measurements were taken at the prescribed time more often, and glucose control improved in comparison with the preceding paper protocol [25]. Unfortunately, a PDMS plug-in with an undisclosed 4-page flowchart was used, limiting widespread usability, and in our opinion a formula-based approach as taken by GRIP will be easier to optimize and customize to different patient groups than a flowchart-based set of rules. Naturally, in case the latter approach may prove to yield better control, GRIP's advice generation module can be easily altered to follow that approach.

We are currently in the process of starting implementation of GRIP at other ICUs. Furthermore, we are implementing GRIP in the coronary care unit. We consider a personalized algorithm to be the key to more efficient and safe glucose control in acute coronary care.

## Conclusion

GRIP, a computer decision support system for glucose control by intensive insulin therapy, exhibited efficient glucose control without inducing severe hypoglycemia during a 4 month period. Acceptance by nurses was excellent, with minimal training needed. GRIP will be released as free/open source software.

## Competing interests

The author(s) declare that they have no competing interests.

## Authors' contributions

MV is the main designer and developer of the GRIP computer program, and was responsible for instructing nurses, taking questionnaires, and data analysis. FZ helped extending the program to the cardiologic domain. MN co-designed the GRIP program and the advice algorithm and is primary investigator of the project. All authors were involved in drafting the manuscript and agree to its publication.

## Pre-publication history

The pre-publication history for this paper can be accessed here:


